# Half a century of changing mercury levels in Swedish freshwater fish

**DOI:** 10.1007/s13280-014-0564-1

**Published:** 2014-11-15

**Authors:** Staffan Åkerblom, Anders Bignert, Markus Meili, Lars Sonesten, Marcus Sundbom

**Affiliations:** 1 Department of Aquatic Sciences and Assessment, SLU, P.O. Box 7050, 750 07 Uppsala, Sweden; 2 Department of Environmental Research and Monitoring, Swedish Museum of Natural History, P.O. Box 50007, 104 05 Stockholm, Sweden; 3 Department of Applied Environmental Science (ITM), Stockholm University, 106 91 Stockholm, Sweden

**Keywords:** Mercury, Freshwater, Environmental Quality Standard, Trend analysis

## Abstract

The variability of mercury (Hg) levels in Swedish freshwater fish during almost 50 years was assessed based on a compilation of 44 927 observations from 2881 waters. To obtain comparable values, individual Hg concentrations of fish from any species and of any size were normalized to correspond to a standard 1-kg pike [median: 0.69 mg kg^−1^ wet weight (ww), mean ± SD: 0.84 ± 0.67 mg kg^−1^ ww]. The EU Environmental Quality Standard of 0.02 mg kg^−1^ was exceeded in all waters, while the guideline set by FAO/WHO for Hg levels in fish used for human consumption (0.5–1.0 mg kg^−1^) was exceeded in 52.5 % of Swedish waters after 2000. Different trend analysis approaches indicated an overall long-term decline of at least 20 % during 1965–2012 but trends did not follow any consistent regional pattern. During the latest decade (2003–2012), however, a spatial gradient has emerged with decreasing trends predominating in southwestern Sweden.

## Introduction

High mercury (Hg) levels in the environment have been of concern in Sweden since they were recognized during the late 1950s in dead birds contaminated by fungicide-treated seed (Borg 1959; Borg et al. 1965), and during the 1960s also in freshwater fish in remote areas (Johnels et al. 1967; Ackefors 1971). Freshwater fish is a dominant source of Hg to birds and mammals feeding on fish (Basu et al. 2005; Evers et al. 2008). Human intake of Hg from predatory fish and other aquatic foods is considered a serious exposure route (NFA 2012). Human hair Hg levels, a good indicator of Hg exposure to humans, are positively correlated to the consumption frequency of freshwater fish in Swedish angling societies (Johnsson et al. 2004). The presence of organic pollutants might have an ecotoxicological effect on wildlife that makes the effect from Hg difficult to distinguish. For instance, an increase in Swedish otter populations between 1968 and 1999 coincided with a decrease in otter polychlorinated biphenyl (PCB) concentrations but did not relate to an apparent increase in Hg concentrations in otters (Roos et al. 2001). The exposure of Hg from small fish species to piscivorous birds during egg laying and hatching was significant compared to other periods (Eagles-Smith and Ackerman 2009). This imposes that selection of size and species for monitoring purposes, and also timing for field collection, needs to be considered in the strategic design of monitoring programs.

While total Hg is usually analyzed and reported in environmental monitoring programs, it is its organic form, methyl-Hg^+^ (MeHg), that biomagnifies and exerts serious neurotoxic effects on humans and wildlife (Chan et al. 2003; Mergler et al. 2007; Scheuhammer et al. 2007). The proportion of MeHg relative total Hg increases in the food web, from 15 % in phytoplankton to >95 % in fish (Watras and Bloom 1992). Ecologically relevant toxicity thresholds for dietary exposure of MeHg in fish have been proposed in the range 0.10–0.40 mg kg^−1^ wet weight (ww) (cf. Depew et al. 2012). To prevent potential negative ecological effects in fish-feeding communities, an assessment factor of 10 is applied to derive toxic thresholds, i.e., Environmental Quality Standard (EQS) (EC 2011). The EQS for Hg in freshwater biota is by this approach set to 0.02 mg Hg kg^−1^ ww by the EU Water Framework Directive (WFD) (EP 2008). Based on risk assessments and other societal considerations, the Food and Agriculture Organization/World Health Organization (FAO/WHO) (CA 1995) and European Commission (EC 2006) use advisory levels of 0.5–1.0 mg MeHg kg^−1^ ww in fish that are considered safe for human consumption. Considering these health advisory guidelines and in the light of current levels in the environment, the ability to detect trends and understand processes from existing datasets is crucial in the evaluation of societal actions with the aim of reducing Hg levels in freshwater biota.

Measures to combat consequences related to high Hg concentrations in freshwater fish have been deployed in Sweden by various means, e.g., prohibiting consumption of fish from targeted lakes and health advisory guidelines by the Swedish National Food Agency (Ohlin 1982; Björklund et al. 1984; Petersson-Grawé et al. 2007; NFA 2012). As Hg contamination became recognized as a transboundary and global problem, international agreements have been implemented with the aim to reduce fish Hg concentrations, i.e., critical load estimations for the convention on long-range transboundary air-pollution (CLRTAP 2003) and classification of water bodies based on the WFD EQS (EP 2000). In November 2013, the Minamata Convention on Mercury was signed by 93 countries with the aim of globally protecting human health and the environment from the adverse effects of Hg (UNEP 2013). Monitoring programs are needed to evaluate the effects of environmental policies and societal management activities with the aim of supporting national and global treaties, and to establish recommendations to protect human health and ecosystems from the effects from Hg contamination. In this context, the availability of long-term monitoring data of Hg from freshwater ecosystems is required to evaluate if measures taken to decrease exposure of Hg to humans and wildlife are efficient.

The challenge in detection of temporal trends is to separate non-temporal variance within and among lakes (e.g., effects of trophic magnification, growth rate or body size) from spatiotemporal patterns in fish Hg levels (e.g., Johnels et al. 1967; Lindqvist et al. 1991; Watras et al. 1998; Lavoie et al. 2013). Non-temporal variance often contributes most to variability and operates both within and among lakes (Sonesten 2003a). To overcome the effect of a size-Hg relationship, normalization to a standard 1-kg pike has long been applied for comparison of fish Hg concentrations across lakes and regions and over time (Olsson 1976; Håkanson et al. 1988; Meili et al. 2004a, b). To transform fish Hg concentrations to correspond to a standard 1-kg pike, various techniques have been applied, such as dividing the Hg concentration by the fish weight (Johnels et al. 1967; Björklund et al. 1984; Sonesten 2003a), selection of fish Hg data within specific size ranges (Sonesten 2003a; Åkerblom et al. 2012), linear regression on weight (Håkanson et al. 1988; Lindqvist et al. 1991), or individual normalization using non-linear species-specific transfer functions (Meili et al. 2004a, b; Munthe et al. 2004). The latter permits the normalization of fish Hg data both across fish species and on an individual basis without assumptions of linearity, for example, to generate a standard 1-kg pike value also for lakes without pike. Temporal trends in fish Hg concentrations have previously been assessed statistically by means of linear regressions (Håkanson et al. 1988; Johansson et al. 2001; Miller et al. 2013), *t* tests (Miller et al. 2013), and *z* tests of proportions of lakes (Åkerblom et al. 2012), but all these methods have limitations in describing non-linear changes and fluctuations. An application of generalized additive models (GAMs) provides the opportunity to capture non-linear trends and allow for the estimation of underlying trends in environmental data (Wood 2011).

The aim of this study was to explore spatial and temporal patterns of Hg concentrations in freshwater fish in Sweden over the past 50 years. Hg concentrations in Swedish freshwater biota have been collected regularly since the late 1960s, and are in care by the national data host (www.ivl.se), commissioned by the Swedish Environmental Protection Agency (www.swedishepa.se). The data from Swedish monitoring programs of Hg in freshwater biota include several fish species (mainly northern pike (*Esox lucius*), Eurasian perch (*Perca fluviatilis*), and Arctic charr (*Salvelinus alpinus*); Table 1) and lake types with varying capacity for trophic transfer of Hg. This study will address spatial patterns and temporal trends in fish Hg concentrations to be discerned after almost 50 years of collecting Hg data in Swedish freshwater fish, in particular the most recent temporal trends (2003–2012) based on systematic annual monitoring. We have used three approaches here. First, the spatial and temporal pattern for the entire studied period is described using the whole dataset normalized to a standard 1-kg pike. Second, long-term changes as well as their spatial patterns are studied using a subset of resampled waters (lakes and streams/rivers) that have been sampled during two time periods: 1965–1990 and 1991–2012. Finally, trends during the latest decade are analyzed using a subset of annually sampled lakes from the national monitoring program. Apart from these approaches, we will provide an overview of available and public data derived from Swedish Hg monitoring and survey efforts over 50 years. The geographic coverage of Swedish data will be compared with that of fish Hg databases in Canada and the USA.

**Table 1:** Overview of the content of the database compiled for this study: fish species and their number of samples and lakes with Hg concentrations and first and last year of reported data

Fish species	No. of specimens	No. of lakes (Catch per lake)	Hg (mg kg^−1^ ww)				First/last year
			Mean^a^		Median	Quantiles	
			AM	GM		10/90	
Northern pike (*Esox lucius*)	26 823	2649 (10.1)	0.77	0.64	0.68	0.28/1.30	1966/2012
Perch (*Perca fluviatilis*)	16 287	484 (33.7)	0.27	0.19	0.18	0.07/0.57	1968/2012
Arctic charr (*Salvelinus alpinus*)	625	17 (36.8)	0.13	0.08	0.09	0.03/0.34	1965/2012
Roach (*Rutilus rutilus*)	823	28 (29.4)	0.27	0.22	0.21	0.10/0.52	1975/2011
Lake trout (*Salmo trutta*)	144	19 (7.6)	0.22	0.15	0.16	0.03/0.49	1982/2012
Other (10 species)	225	36 (6.3)	0.23	0.15	0.14	0.05/0.44	1969/2012

^a^Mean of Hg concentrations presented as arithmetic mean (AM) and geometric mean (GM)

## Materials and methods

### Data acquisition

The bulk of data used in this study was retrieved from the Swedish national data host, Swedish Environmental Research Institute (www.ivl.se), which is contracted to store all records on Hg and other environmentally hazardous substances in biota collected within national and regional monitoring programs and surveys. Strategies for data collection range from carefully designed monitoring programs bound by long-term commitments aimed for environmental assessment to screening initiatives focusing on human consumption of freshwater fish. The former category belongs to the national monitoring program for Hg in freshwater fish, which is coordinated by the Swedish Museum of Natural History (SMNH) that supports the annual collection and analysis of perch, pike, and Arctic charr from a total of 32 lakes (SMNH 2011). Another national program, maintained by Integrated Studies of the Effects of Liming Acidified Waters (ISELAW), has followed Hg concentrations in young perch in a total of 23 limed and acidic lakes (Sundbom 2009). Waters and samples of fish Hg outside the two nationally coordinated programs were classified as regionally coordinated survey programs. To close identified gaps in the dataset that was downloaded directly from the data host (on December 11, 2013), we collected data from the main Hg analysis laboratory involved in national and other fish monitoring (ITM, Stockholm University). These included newer data not yet reported to the data host as well as Hg data from older national, regional, or research programs. Non-fish biota and duplicate records were excluded from the dataset before further analysis.

### Transformations and statistics

To improve comparability of Hg concentrations among fish of different size and species, and among sites in space and time, observed individual Hg concentrations ([Hg]_obs_) were standardized to correspond to a 1-kg pike in the same lake ([Hg]_std_), based on an empirically supported transfer function applicable to any fish species at any site, as described in a UN/ECE manual (Meili et al. 2004b):

$$ \left[ {\text{Hg}} \right]_{\text{std}} = \left[ {\text{Hg}} \right]_{\text{obs}} /\left( {f_{\text{HgY}} + f_{\text{HgW}} \,W^{2/3} } \right), $$

where W is the fish body weight in kg (taken as a unitless value), f_HgY_ a parameter representing the concentration ratio between newly hatched young fish and 1-kg pike, and f_HgW_ a species-specific empirical coefficient. Parameters were set to default values (Meili et al. 2004b) derived from a large database (Munthe et al. 2004), except for a minor adaptation for large perch (based on Meili et al. 2004a), and a representative bulk value for rare individuals of other species (cf. Meili et al. 2004b): f_HgY_ to 0.13 and f_HgW_ to 0.87 for pike, 1.65 for perch, and 1 for other species. Additionally, for the evaluation of recent trends in national monitoring lakes, where large amounts of single-species data allow for a stable site-specific normalization, log Hg concentrations were normalized by ANCOVA using fish body length and age as covariates prior to trend estimation by linear regression on the residuals from the ANCOVA model.

Several approaches were used to analyze temporal trends and spatial patterns. First, linear regression and GAM were fitted on log-transformed and normalized (1-kg pike) Hg concentrations ([Hg]_std_). GAM is useful for analyzing non-linear relationships by combining the ease of interpretation of linear models with the flexibility of nonparametric smoothing techniques. Here, a simple GAM with Hg as response variable and a smoothing function of time as the single explanatory variable was fitted to (1) all data, (2) subset of lime-treated lakes, and (3) a subset of non-limed lakes. A lake was classified as limed if it had been subject to liming at any time during the study period according to the national liming database (http://kalkdatabasen.lansstyrelsen.se). The GAMs were fitted using R (package mgcv 1.7–28, Wood 2011) with default settings (link function: identity; family: gaussian) and cross validation to determine the optimal degree of smoothing. A second approach using a paired *t* test was applied to compare Hg concentrations in fish from resampled waters between two time periods (1965–1990 and 1991–2012). Finally, on the same subset of matched lakes, spatial trend surface tests were employed with the spatiostatistical software TISS (Thematic Images and Spatial Statistics), and all descriptive statistics regression and ANCOVA analysis were assisted by JMP 11.0 (SAS Institute Inc., Cary, NC, USA). The significance level was set to 5 % for all tests.

## Results and discussion

### Swedish fish Hg database

The extended database contained 44 927 observations on fish Hg concentrations from 15 species in 2881 waters in Sweden (Table 1). The dataset was based mainly (>99.2 %) on Hg in fish muscle tissue from pike (59.7 %), perch (36.2 %), roach (*Rutilus rutilus*, 1.8 %), and Arctic charr (1.4 %). For the major fish species, the highest median Hg concentrations were found in pike, followed by roach, perch, and Arctic charr (Table 1). Hg concentrations in roach are usually lower than in perch (e.g., Meili 1991; Sharma et al. 2008). Within few lakes (*n* = 11) in which roach have been sampled along with perch, the Hg concentrations in perch were higher compared to roach (data not shown). The order in which Hg concentrations differed between fish (Table 1) was thus partly an effect of skewness in sampled waters, species and fish size there in.

**Fig. 1:**
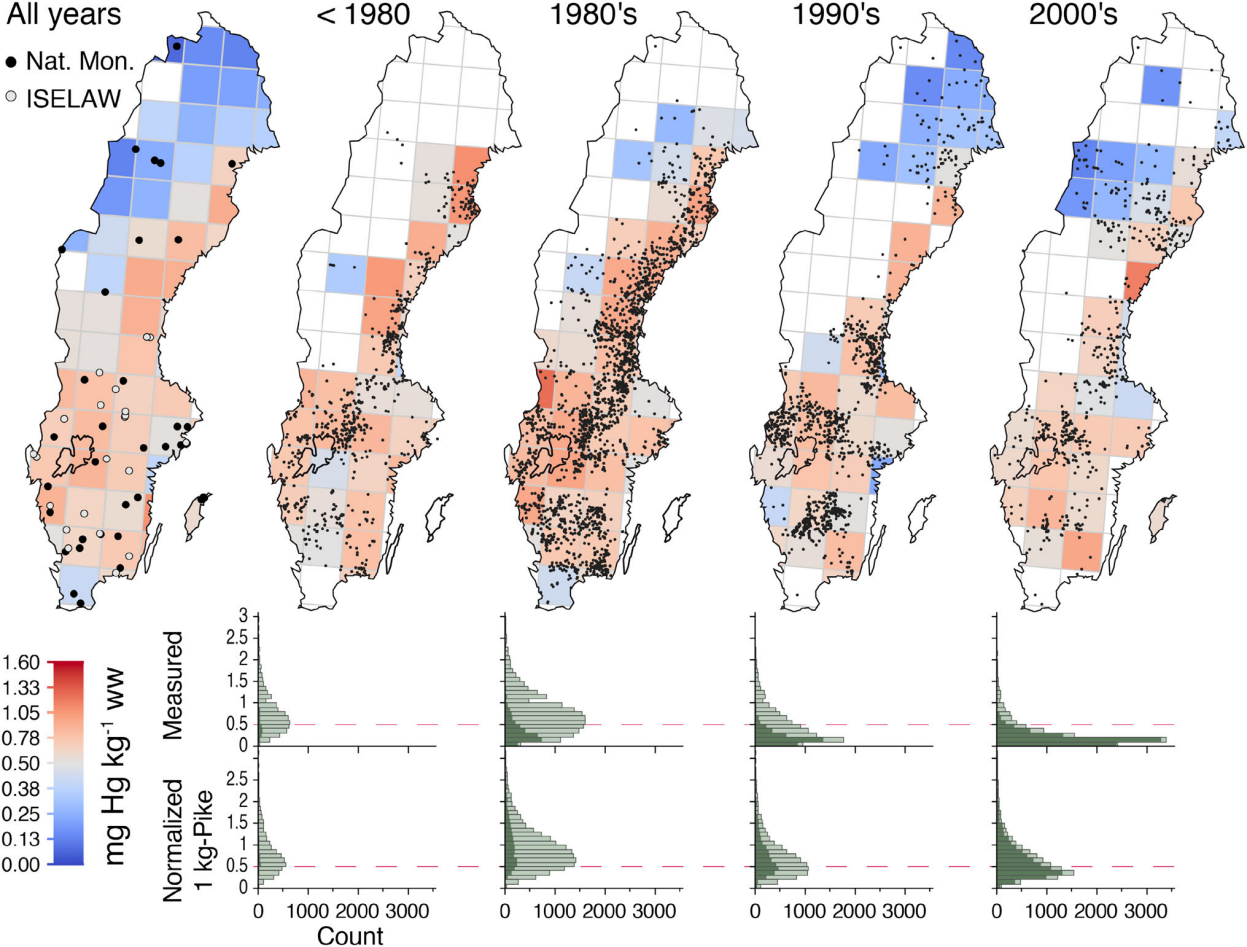
Distribution of fish total ww Hg concentrations (mg Hg kg^−1^ ww) in Swedish lakes over five decades. The map color represents the mean of lake means within a grid cell (European Environment Agency reference grid, 100 km). Lake means were obtained after normalizing individual Hg concentrations in fish of all species and sizes to represent Hg in a typical 1-kg pike (see “Materials and methods” section). The *leftmost map* is based on all data from 1965 to 2012; 42 000 individual fish from 2655 waters, including the lakes within the national monitoring and ISELAW programs (position shown in map). The four maps to the* right* illustrate the spatial distribution of sampling efforts (*dots* representing lakes sampled) and Hg concentrations during different time periods. Histograms show distributions of pike and perch Hg concentration before (*upper row*) and after normalization to 1-kg pike. The *red reference lines* denote the EU and FAO/WHO health advisory guideline for Hg in fish as food

Transfer functions normalizing Hg concentrations to represent Hg in a typical 1-kg pike was applied to overcome skewnesses in differences in collected species between waters, regions and time periods, and fish size. The application of transfer function was possible for 42 000 data points in the dataset with a mean ± SD [Hg]_std_ of 0.84 ± 0.67 mg kg^−1^ ww (median = 0.69 mg kg^−1^ ww) (Fig. 1). In order to test the performance of the transfer function, we identified 200 instances where Hg had been determined in both perch and pike from the same lake within a month’s time. Observed Hg concentrations were on average 24 % higher in pike than in perch (paired *t* test on log Hg, *p* < 0.001, *n* = 200). After normalization to 1-kg pike, this difference between sympatric pike (median length = 46 cm) and perch (median length = 21 cm) narrowed down to only 0.9 % and was not statistically significant (paired *t* test on log Hg 1-kg pike, *p* = 0.76). The transfer function was also successful in dampening the Hg-size relationship within species. The regression slope for log Hg vs fish length was calculated for 894 subsets of perch and 334 subsets of pike, each with >10 individuals caught within the same month and lake. The median slope (% per cm) decreased from 7.8 to 0.2 in perch and from 2.9 to −0.3 in pike after the transfer function was applied. Thus, the transfer function proved to adequately account for typical relationships between Hg concentrations and both body size and fish species, based on a minimum of preset parameters.

Of the two major fish species in the database, the median sample size of the number of fish from the same year and lake individually analyzed for Hg was 5 for pike (min–max: 1–170) and 10 for perch (1–76). 1427 waters were sampled only once within a single year, while 1228 waters were sampled more than 1 year. The median length of the time series from the repeatedly sampled waters was 9 years. A total of 246 waters had time series extending between 20 and 45 years. The lake with the longest annual time series, Lake Stensjön in Tyresta National Park, has been analyzed for 36 consecutive years between 1977 and 2012.

The relative number of reported specimens and waters per area was estimated for the Swedish database based on the total area of Swedish land and water and the area of water (Table 2). The relative density of specimen per unit water and land area in Sweden was considerably higher compared to fish Hg databases from Canada, covering 40 years with reported data from various monitoring and research programs (Depew et al. 2013) and databases on fish Hg concentrations collected by federal, state, and local agencies in the USA (Chalmers et al. 2011). Databases on Hg in fish from the Great Lakes region (Monson et al. 2011) and Ontario (Gandhi et al. 2014) also withhold compilations on Hg in fish from a large, densely populated region, and were included for comparison of fish Hg monitoring efforts. The North American studies were based on regional and national databases and considered comparable to the database in this study. This type of comparison gives an indication of the efforts between countries to perform fish Hg monitoring. It is important; however, to note that it does not justify the database quality since the possibility for evaluation of spatial or temporal trends depends on the representativeness of sampled waters and fish species within individual studies.

**Table 2:** The relative number of reported individual samples and lakes per km^−2^ land and water in reported fish Hg databases from Sweden (this study), Canada (Depew et al. 2013), USA (Chalmers et al. 2011), Ontario (Gandhi et al. 2014), and the Great Lakes region (Monson et al. 2011)

Country	Total land area (km^2^)	Total water area (km^2^)^a^	Reported fish individual samples/lakes	Relative number of reported individual samples per land area/water area (km^−2^)	Relative number of reported lakes per land area/water area (km^−2^)
Sweden	40 7340	40 080	44 927/2881	0.110/1.12	0.0071/0.072
Canada	9 093 507	891 163	330 000/5000	0.036/0.37	0.0005/0.006
USA	9 147 593	377 768	74 867/7759	0.008/0.20	0.0008/0.021
Ontario, Canada	917 741	158 654	97 888	0.107/0.62	
Great Lakes	2 400 000	350 136	63 872	0.003/0.18	

^a^Total land and water area for Sweden, Canada, and USA retrieved 8 July, 2014 from http://www.scb.se/statistik/MI/MI0802/2012A01/MI0802_2012A01_SM_MI65SM1201.pdf, http://www.statcan.gc.ca/tables-tableaux/sum-som/l01/cst01/phys01-eng.htm and http://www.census.gov/geo/reference/state-area.html, respectively

### Strategies of Swedish freshwater monitoring of Hg in biota

**Fig. 2:**
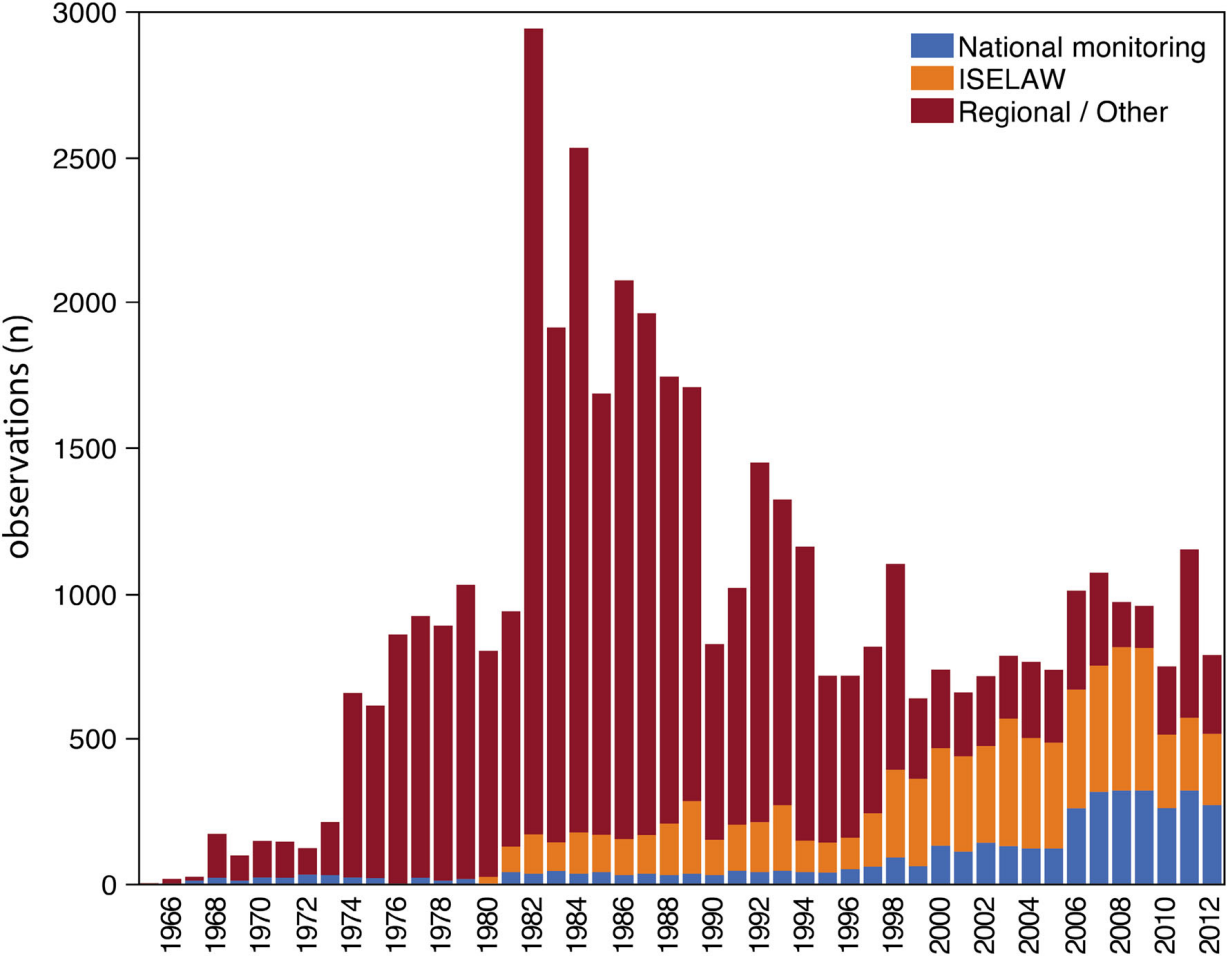
Reported observations from national monitoring, ISELAW, and regional monitoring/survey or other studies

The majority of waters and specimens within the national database originated from regional and local surveys and monitoring (Fig. 2). The strategy and coordination between national and local monitoring activities have, however, changed over time. The percentages of waters and number of specimens in nationally relative regionally coordinated monitoring activities between 1970 and 2000 were 1 and 12 %, respectively. A significant change in these proportions was seen after 2000 as the proportions of waters and number of specimens in national and regional monitoring increased to 11 and 68 %, respectively (Fig. 2). The longest nationally coordinated monitoring of Hg in freshwater fish has been conducted in lakes Bolmen and Storvindeln, from which dorsal muscle samples from northern pike have been collected since 1967 (SMNH 2011). Collection and analysis of Hg in perch from another 27 lakes have also been included between 1997 and 2007 in nationally coordinated programs. There has also been a shift in the target species used for monitoring of Hg in aquatic biota (Fig. 1). Northern pike was predominating between 1966 and 2000 (89 %), while perch was collected at higher abundances between 2000 and 2012 (69 %) (Fig. 1). The largest number of waters with fish Hg data in the database was reported between 1980 and 1989 (*n* = 2009) followed by a decrease in reported waters by almost 50 %, both between 1990 and 2000 (*n* = 1056) and from 2000 until 2012 (*n* = 573). Between 1965 and 1979, fish Hg data from 703 waters were reported to the database. Waters in northern Sweden have been reported more frequently since the 1980s.

### Swedish freshwater fish Hg concentrations compared to EQS

The EQS for Hg in freshwater biota according to the WFD (0.02 mg kg^−1^ ww) was exceeded in all species and waters, except few individuals, within the database (Table 1; Fig. 1). In Arctic charr in Lake Abiskojaure, northernmost Sweden, the Hg concentrations exceeded the EQS manifold (1–35 times). Health advisory guidelines according to the FAO/WHO (0.5–1.0 mg MeHg kg ww) were also exceeded in more than half (52.5 %) of the waters in the database after 2000 (Fig. 1). The potential risk was not evenly distributed among Swedish lake ecosystems and it depended on, besides fish species, lake and catchment characteristics with high fish Hg concentrations in low productive humic lakes within forested catchments (Lindqvist et al. 1991; Sonesten 2003b). The main feature of the geographical pattern for Hg in fish was decreasing levels from south to north with the highest concentrations found in central Sweden and along the Swedish coast of the Gulf of Bothnia (Fig. 1). The elevated fish Hg concentrations in these areas may be influenced by historical emissions of Hg to both water and air as suggested by Lindqvist et al. (1991). However, ecosystem-specific characteristics also contributed to the variability in fish Hg concentrations between regions. This was also true for the southernmost part of Sweden, where low Hg concentrations were found despite a large burden from atmospheric deposition. These areas are mainly agricultural and the freshwater bodies mainly eutrophic, which may decrease Hg concentrations at higher trophic levels due to less Hg in diet (bio-dilution; Pickhardt et al. 2002) and higher growth rates (growth dilution; Verta 1990; Karimi et al. 2007).

The response of Hg concentrations in freshwater fish to changes in anthropogenic Hg emissions depends on factors affecting Hg cycling and bioaccumulation (e.g., catchment characteristics, water quality, trophic structure, and climate) (Munthe et al. 2007). The sensitivity of individual waters of transferring atmospheric Hg deposition to fish thus varies considerably (Harris et al. 2007; Orihel et al. 2007; Corbitt et al. 2011). The efforts to decrease Hg emissions by international agreements should assist freshwater ecosystems to recover from past Hg contamination but will also depend on other factors apart from a decrease in Hg emissions.

### Spatial and temporal changes

#### Large-scale changes

**Fig. 3:**
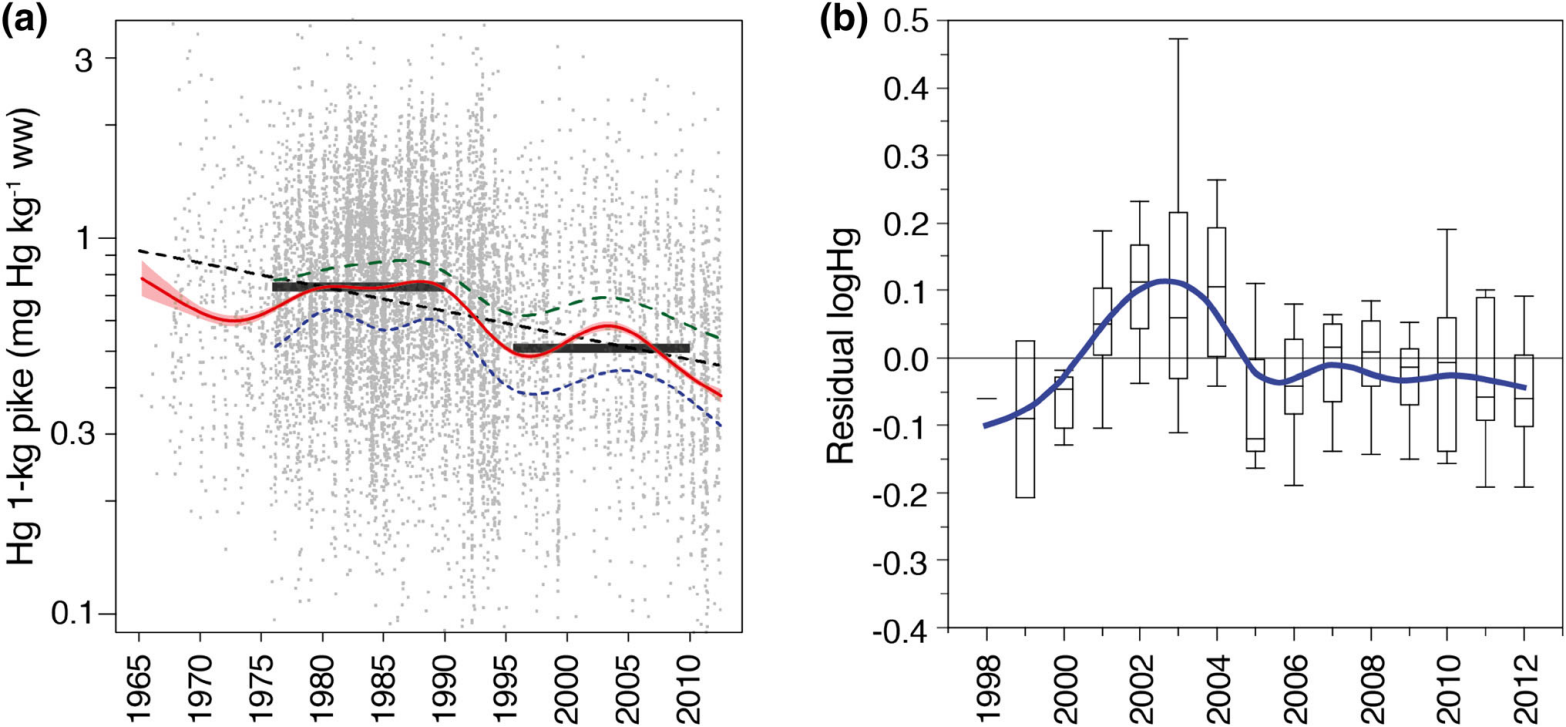
Total Hg concentrations in Swedish fish 1965–2012. **a** Normalized (1-kg pike) Hg concentrations of 10 176 catches; each point represents the mean from a single date and site. A linear regression model (*black dashed line*) and a GAM (*red line* ± SE) were applied to all data to visualize temporal patterns. The *parallel dashed lines* are separate GAMs fitted either to limed lakes (*upper green dashed line*) or to never-limed lakes (*lower blue dashed line*). The *black step lines* indicate the geometric mean Hg concentrations between 1976–1990 (0.74 mg kg^−1^ ww) and 1996–2010 (0.52 mg kg^−1^ ww). **b** Temporal pattern of untransformed Hg concentrations in medium-sized perch (total length 140–220 mm) from 11 national monitoring lakes during 1998–2012 (years 1998 and 1999 only represented by one and two lakes, respectively). Box-plots depict median, 25/75 and 2.5/97.5 percentiles, based on the distribution of residual Hg concentrations, i.e., deviation of annual log Hg from the within-lake mean for the period

Visualization of all the available data in maps and diagrams indicated that large-scale changes in fish Hg concentrations have occured over the last 50 years (Fig. 1). However, changes in intensity (Figs. 1, 2), geographical distribution (Fig. 1), and general objectives of the data collecting efforts require caution when delineating temporal trends. Linear regression based on the normalized concentrations of all fish suggested that Hg concentrations have decreased approximately 1 % per year since 1970 (*R* ^2^ = 0.051, *p* < 0.001), corresponding to a decrease of about 30 % during 40 years (Fig. 3a). A GAM fitted to the same data also indicated a long-term decrease, but following a more complex pattern (deviance explained = 8.3 %, *p* < 0.001). According to the GAM, average Hg concentrations in 1-kg pike equivalents increased during the 1970s and peaked at the end of the 1980s before decreasing sharply between 1990 and 1996. During the late 1990s, Hg levels increased again and, after peaking by 2003, they appeared to have decreased up to present. The pattern of a reversed trend in the late 1990s also appeared for non-transformed Hg concentrations in perch from 11 annually sampled lakes (Fig. 3b). The two 15-year periods 1976–1990 and 1996–2010 appeared to represent two separate states with geometrical means of 0.74 and 0.52 mg Hg kg^−1^ ww, respectively. Thus, most of the observed 30 % decline appeared to have occurred during the relatively short period between 1990 and 1996, while levels remained relatively constant 15 years before and after this transition period (Fig. 3a). Some of the sampled waters before the 1990s may not be representative because of a possible focus on lakes suspected or known to have a comparatively high Hg levels, e.g., locally polluted by point sources (Lindqvist et al. 1991).

Both the long-term decrease and rapid shift in Hg levels between 1976–1990 and 1996–2010 suggested by the GAM may, however, have been influenced by changes in monitoring strategies; for example, a shift from an earlier focus on acidified and lime-treated waters to the present focus on more pristine waters. Liming has been applied to surface waters to restore aquatic ecosystems from effects from acidification. In lakes with effective liming a 10–20 % decline in pike Hg concentrations have been observed (Meili 1995). Lakes in the limed group had on average considerably higher Hg concentration than lakes that were never subject to liming; possibly reflecting that many of these lakes were acidic or acidified. Here, the long-term temporal pattern was, however, quite similar in limed and non-limed waters as suggested by GAMs fitted to each subset of the data (Fig. 3a). Apparently, the shift in strategy from an earlier focus on acidified and lime-treated lakes to a relatively larger focus on pristine waters did not confound the long-term pattern in a major way. The observation here that changes in Hg levels roughly follow the same pattern, irrespective of being based on subsets of normalized but heterogeneous data (Fig. 3a) or national monitoring time series (Fig. 3b), suggests that a common set of large-scale external forcing factors may exist.

To improve comparability over time, we divided the data into two time periods and restricted the data to only include waters that were sampled in both periods. For the periods 1965–1990 and 1991–2012, 514 resampled waters were identified. A comparison between the two periods shows a significant decrease in Hg concentrations (paired *t* test: *p* < 0.001, two-sided, *n* = 514). On average the decrease was 20 %, which was less than, but still comparable to, the 30 % long-term decline found by linear regression and GAM on the extensive but much more heterogeneous dataset (Fig. 3a). A previous study compared Hg concentrations in perch (size adjusted to 200 g/25 cm) between 1974–1995 and 1996–2005 in Sweden that also showed a significant decrease of 21 %, while an increase of about 10 % was found in Finland (Miller et al. 2013). An increase was also found in perch and brown trout collected from southeastern Norway between 1991 and 2008 (Fjeld and Rognerud 2009).

**Fig. 4:**
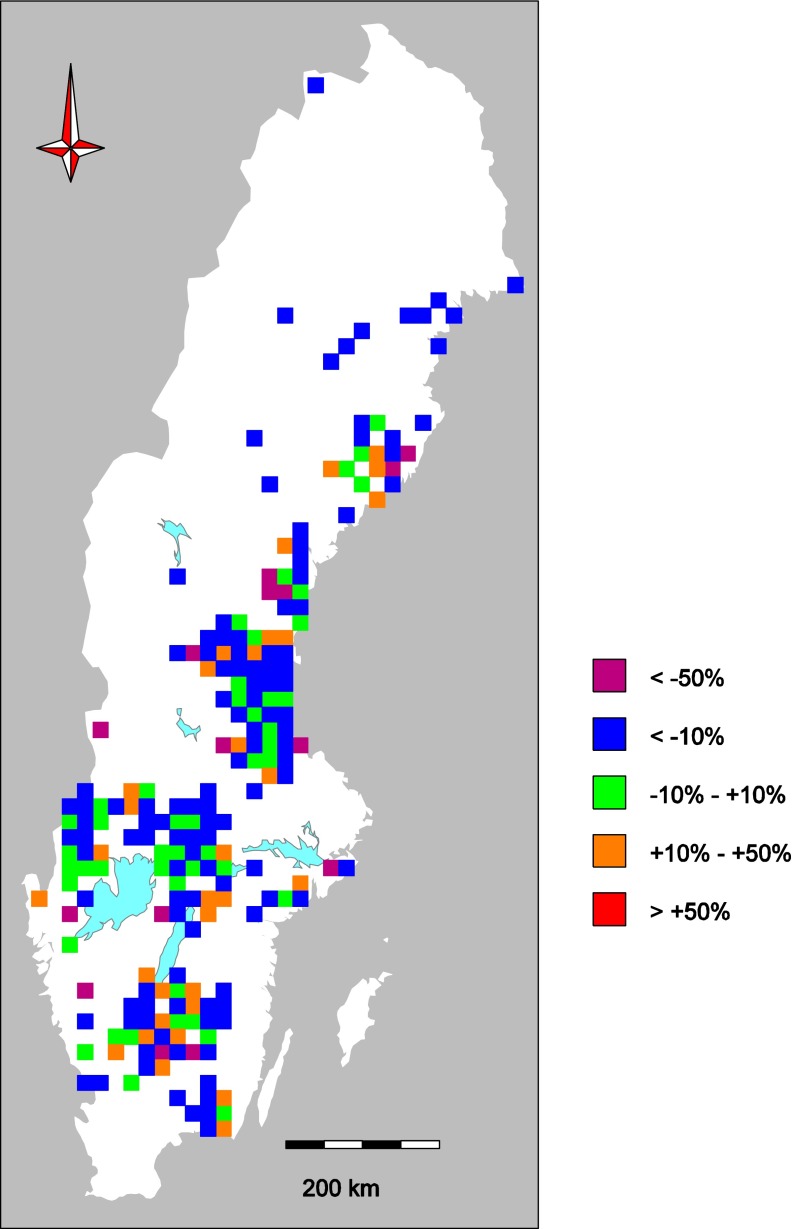
Change in normalized (1-kg pike) total ww Hg concentrations based on 514 resampled lakes visited both 1965–1990 and 1991–2012. Colors show the mean change within 20 × 20 km areas containing at least one resampled site. The average change was −20 %

Regional differences in temporal trends were not evident from the data dominated by our normalized (1-kg pike) Hg concentrations (Fig. 4). Using the 514 resampled waters for spatiotemporal analysis, the current study showed that the change in fish Hg concentration was not evenly distributed. In some regions, we saw increasing Hg concentrations, but it was not possible to show a systematic geographical pattern in time trends using spatial analysis (Fig. 4). In several large areas of Sweden, it was not possible to find waters where Hg analysis were carried out in both periods (cf. Fig. 1).

In previous studies, shorter temporal (decadal) trend analysis of Hg concentration in freshwater fish has identified periods with increasing Hg concentrations as well as decreases in northern pike that fit well with the pattern observed herein. From the beginning of the 1970s until 2010, reported time trends in fish Hg concentration can roughly be divided into three phases. Fish Hg concentrations increased significantly during the 1970s until 1980s, followed by a decrease from the end of the 1980s until the 1990s (Håkanson et al. 1988; Johansson et al. 2001). The third phase has been estimated to last from the end of the 1990s and a decade forth, with pike Hg concentrations tending to increase in Swedish lakes (Åkerblom et al. 2012).

#### Recent trends

After a period of declining Hg levels (Johansson et al. 2001), recent reports about trends from Sweden (Åkerblom et al. 2012), Norway (Rognerud et al. 2009), and North America (French et al. 2006; Monson 2009; Gandhi et al. 2014) of increasing Hg concentrations in fish from the mid 1990s to about 2005 were discouraging. Now, with access to both longer time series and larger numbers of lakes in the national monitoring program, it is possible to further investigate whether the increasing trend persists or if it is a part of a more long-term, multiannual variation as suggested by Fig. 3a, b. The current national monitoring program includes 27 lakes where intermediately sized perch (total length 140–220 mm) are collected annually of which 10 similarly sized individuals are analyzed for Hg, among other contaminants. A few of these lakes have been monitored for decades: about 10 lakes since 1998–2000 with the remainder added in 2005–2007 following the latest major revision of the national monitoring program (SMNH 2011). Individual fish length, weight, and age are routinely determined, which together with the abundance of data allows for a site-specific approach to account for individual and inter-annual variation.

**Fig. 5:**
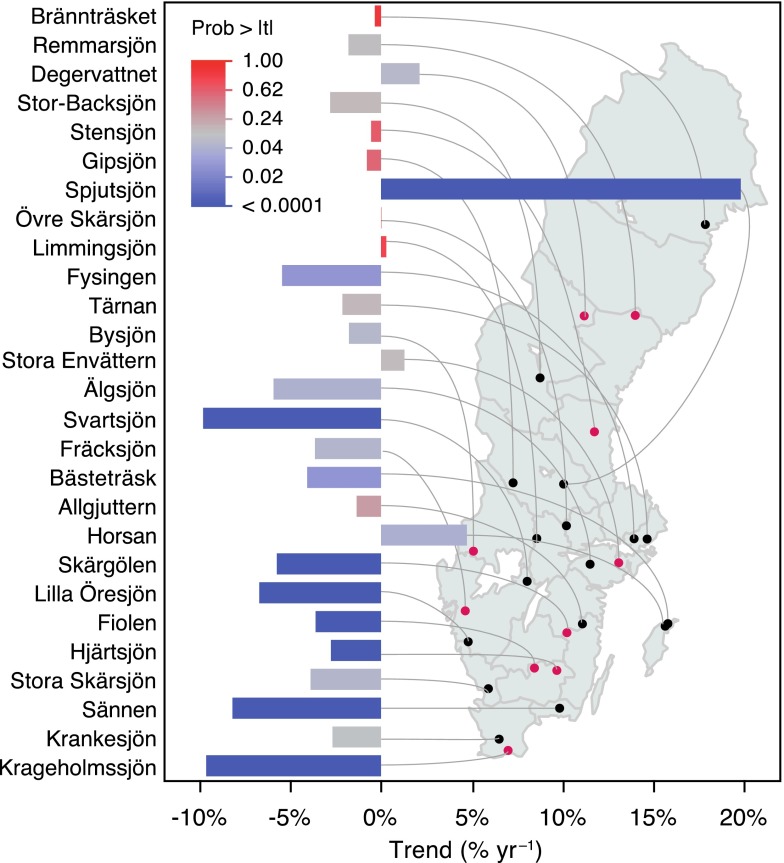
Recent trends of total ww Hg concentrations in medium-sized perch (total length 140–220 mm) from 27 national monitoring lakes during the period 2003 (*red dots*, cf. Fig. 3b) or 2005/2006/2007 (*black dots*) to 2012. The lakes are ordered by latitude. Trends were estimated by linear regression on log-transformed Hg concentration normalized site specifically for fish age and body length. The *bar color* represents the probability of individual trends being equal to zero (*t* test). *Blue bars* highly significant, *red bars* not significant. Note the variable timing with respect to overall fluctuations such as the peak preceding 2005 (Fig. 3b)

The majority of the linear trend estimates were negative during the period of 2003 (or later for some lakes) to 2012, especially in waters in the southwestern part of Sweden where Hg concentrations decreased with up to 10 % per year (Fig. 5). In the northern part, the trends were weaker and mostly not significant. In one lake, however, concentrations increased steeply with about 20 % per year, which may indicate local anthropogenic sources of Hg (Fig. 5). Although this short-term trend analysis using 6–10 years of high-quality and consistent data suggests that Hg in fish is decreasing (again), it is not possible to conclude that this situation will persist, given the complex long-term dynamics discussed above.

## Conclusions

Efforts to collect fishes, analyze samples, and report and collect fish Hg data over nearly 50 years enabled derivation of spatial and temporal patterns of Hg levels in Swedish freshwater fish. Fish Hg concentrations in Swedish waters are decreasing by approximately 1 % per year between since 1970. The long-term temporal trends did not show any systematic geographical pattern, despite heterogeneity in controlling processes, such as historically changing atmospheric inputs of Hg (larger decrease in Hg loads in the south compared to the north of Sweden), catchment characteristics, and land use between regions. Despite decreasing trends, fish Hg concentrations are still high (>0.5 mg kg ww) in several regions and health advisory guidelines for human consumption of freshwater fish are still relevant.

The coupling between aquatic species Hg concentrations and potential negative effects on ecosystem functioning depends on relevant biological indicators that can assist in the formulation of EQS for aquatic ecosystems. Variation in target species and size for bird and mammal as well as human consumption habits emphasizes the need for various target species in environmental monitoring strategies to protect ecological and human health according to criteria set by the WFD and WHO/FAO. The violation of the WFD EQS (0.02 mg kg^−1^ ww), even in remote and pristine waters, requires efforts to introduce measures from society to counteract possible negative effects on ecosystem health.

The observations made here, that inter-annual changes appear to happen synchronously to some extent (cf. Fig. 3b), are useful for future efforts in evaluation of the efforts to decrease emissions and use of Hg in products, which is the goal for the Minamata treaty (AMAP/UNEP 2013). The evaluation of source-receptor relationship between Hg emissions and Hg levels in freshwater ecosystems (cf. Meili et al. 2003) requires interdisciplinary research to determine the efficiency of international agreements (i.e., the Minamata treaty). Observations of direct and indirect consequences from reductions in Hg emissions in relation to Hg accumulation in freshwater ecosystems depend on continued monitoring of Hg levels in freshwater ecosystems within Sweden but also across nations and continents.
